# The state of the art in Blue Amazon biodiversity: Protocol for a systematic review

**DOI:** 10.1016/j.mex.2025.103561

**Published:** 2025-08-09

**Authors:** G.B. Farias, M. Bassoi, J.B. Vasconcelos, Y. Costa, J.N. Freire, J. Lucatelli, M. Arangüena-Proaño, A.J. Silva, M. Previero, L.H. Bordin, B.P. Ferreira, L.C. Lopes, E.R. Secchi

**Affiliations:** a Instituto Nacional de Ciência e Tecnologia da Biodiversidade da Amazônia Azul (INCT-BAA). FURG, Rio Grande, Rio Grande do Sul , 96203-900, Brazil.; b Instituto de Oceanografia, Universidade Federal do Rio Grande (FURG). Rio Grande, Rio Grande do Sul 96203-900, Brazil; c Departamento de Fisiologia e Comportamento, Universidade Federal do Rio Grande do Norte (UFRN), Natal, Rio Grande do Norte 59078-970, Brazil; d Departamento de Oceanografia, Universidade Federal de Pernambuco (UFPE). Recife, Pernambuco 50670-420, Brazil.; e Instituto Socioambiental e dos Recursos Hídricos (ISARH). UFRA, Belém, Pará 66077-830, Brazil.; f Laboratório de Ecologia Marinha e Oceanografia Pesqueira da Amazônia, Universidade Federal Rural da Amazônia (UFRA). Belém, Pará 66077-830, Brazil.; g Laboratório de Aves Aquáticas e Tartarugas Marinhas, Universidade Federal do Rio Grande (LAATM-FURG). Rio Grande, Rio Grande do Sul 96203-900, Brazil; h Laboratório de Ecologia Bentônica, Universidade Federal da Bahia (LEB-UFBA), Salvador, Bahia 40170-115, Brazil.; i Laboratório de Ecologia do Plâncton, Universidade Federal Rural de Pernambuco (LEPLANC-UFRPE), Recife, Pernambuco 55810-700, Brazil.

**Keywords:** Marine biodiversity, Brazilian Exclusive Economic Zone, Systematic Literature Review, PRISMA protocol, Marine Conservation

## Abstract

Brazil holds approximately 40 % of its territory in the sea, an area known as the "Blue Amazon" due to its dimension, and strategic, economic and biological significance. Despite this importance, much of Brazil’s marine biodiversity remains poorly mapped and quantified. Therefore, the National Institute of Science and Technology for the Blue Amazon Biodiversity (INCT-BAA) was established to expand knowledge on this region. One of INCT-BAA's primary objectives is to develop an integrated database that combines historical and contemporary information on marine biodiversity. To support this objective, a state-of-the-art systematic literature review was conducted to synthesize existing knowledge on species occurrence, spatial and temporal distribution, natural and anthropogenic threats, ecological scope of studies, and knowledge gaps and trends. Given the broad scope of the review, a specific protocol was developed, requiring adaptations to traditional systematic review methodologies (e.g., elaboration of a robust glossary of categories and methods to extract species locations from papers), which are detailed in this document. The review was conducted over one year using 1,034 search strings across Web of Science, ScienceDirect, and Google Scholar and retrieving over 200,000 species occurrences from 7,687 studies, covering more than 27,000 species from 1917 to 2023.

## Highlights

• A systematic literature review was conducted to synthesize existing knowledge on the biodiversity of the Blue Amazon.
• A comprehensive set of information was extracted from peer-reviewed studies on marine research in the Brazilian marine areas, covering the period from the earliest studies to 2023.
• A database was constructed to support future research and conservation strategies.

## Graphical abstract

**Figure:**
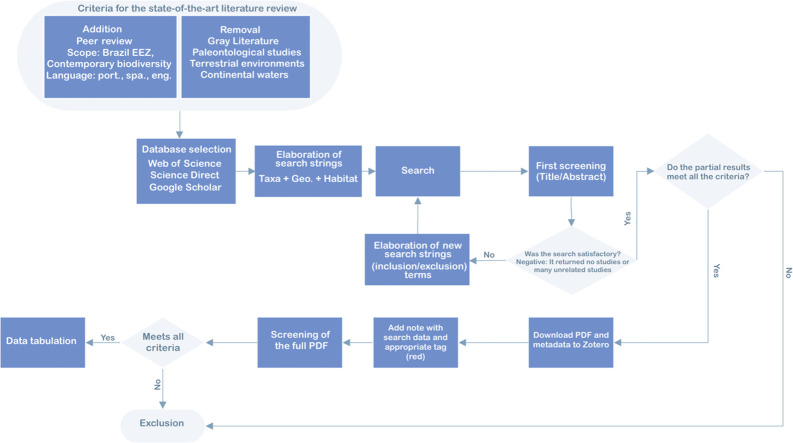

Specifications table

**Table:** 

**Subject area**	Agricultural and Biological Sciences
**More specific subject area**	Marine Sciences
**Name of your method**	Marine Biodiversity Systematic Review Protocol
**Name and reference of original method**	Bramer, W. M., G. B. de Jonge, M. L. Rethlefsen, F. Mast, & J. Kleijnen, 2018. A systematic approach to searching: an efficient and complete method to develop literature searches. **Journal of the Medical Library Association** : JMLA 106: 531–541. Page, M. J., J. E. McKenzie, P. M. Bossuyt, I. Boutron, T. C. Hoffmann, C. D. Mulrow, L. Shamseer, J. M. Tetzlaff, E. A. Akl, S. E. Brennan, R. Chou, J. Glanville, J. M. Grimshaw, A. Hróbjartsson, M. M. Lalu, T. Li, E. W. Loder, E. Mayo-Wilson, S. McDonald, L. A. McGuinness, L. A. Stewart, J. Thomas, A. C. Tricco, V. A. Welch, P. Whiting, & D. Moher, 2021. The PRISMA 2020 statement: an updated guideline for reporting systematic reviews. **BMJ British Medical Journal Publishing Group** 372: n71.
**Resource availability**	Zotero (https://www.zotero.org) OpenRefine (https://openrefine.org)

## Background

**Fig. 1:**
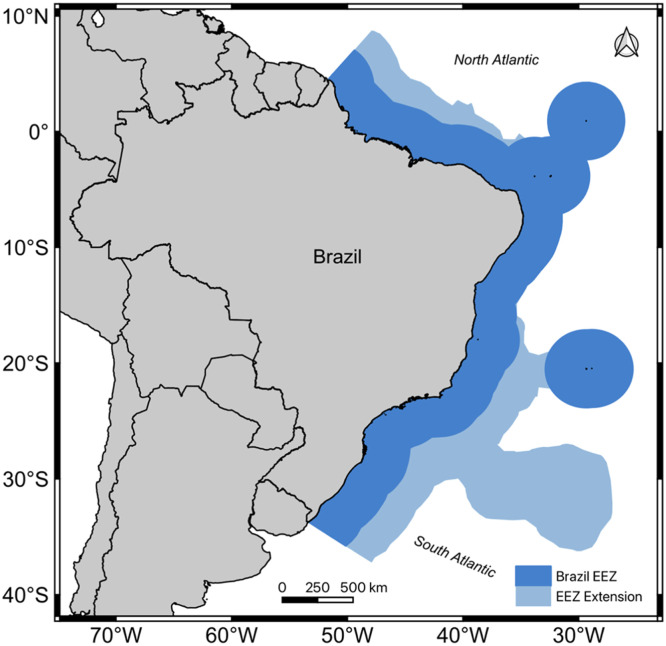
Area of the Brazilian exclusive economic zone (EEZ, Blue Amazon) whose biodiversity studies are considered in the state-of-the-art review.

Brazil has a large marine economic exclusive zone (EEZ) that corresponds to around 5.7 million square kilometers (Fig. 1), an area known as the "Blue Amazon" due to its vast dimension, and strategic and economic importance and biological significance comparable to the Brazilian Amazonia [1]. However, much of this marine biodiversity remains unmapped and unquantified. This biological diversity represents a critical resource for food security, through fishing and aquaculture, and offers substantial biotechnological potential (e.g., new pharmaceuticals, biofuels, dietary supplements) to address various contemporary challenges [2,3]. Conserving this biodiversity increases the resilience of coastal and marine ecosystems against both natural and anthropogenic impacts, including climate change [4].

Concern over the sustainable use of the ocean has been growing globally, and concrete actions to mitigate threats are expected by the end of 2030, as part of the United Nations-declared Decade of Ocean Science [5]. During this decade (2021–2030), it is expected that Brazil will advance in knowledge and produce information to support the conservation of the "Blue Amazon" biodiversity and harness its full potential for national interests. To this end, the National Institute of Science and Technology – Blue Amazon Biodiversity (INCT-BAA, *from the Portuguese*) has been established to create a broad national network of researchers and research institutions, as well as international collaborators from various fields of knowledge [6]. The objectives of INCT-BAA include both basic and applied research on marine biodiversity. In this context, the mapping and understanding of the richness and biological diversity of Brazil’s marine environment by INCT-BAA may bring significant socioeconomic and environmental benefits to the country, supporting the development of public policies for the sustainable use of marine resources.

Among the many objectives of INCT-BAA, two key ones are: (1) to develop a comprehensive database of species occurrences that aggregates both historical and contemporary information, and (2) to promote data integration, analysis, and synthesis to generate effective management recommendations, high-impact scientific contributions, and science communication for society.

To achieve these goals, a state-of-the-art literature review was conducted. The main objective of this state-of-the-art literature review was to comprehensively assess the current knowledge on the biodiversity of the Blue Amazon. It encompasses multiple aspects, including the occurrence of different biological species, their spatial and temporal distribution patterns, threats faced by biodiversity in the Blue Amazon, the ecological scope of the studies in the region, as well as accumulated knowledge and research gaps. This synthesized information will provide a robust foundation for planning future research initiatives and developing effective conservation strategies.

To assess the state of knowledge, the following questions guided the review:

(1) Which species occur in the Blue Amazon?
(2) How is marine biodiversity distributed in the Blue Amazon (e.g., are there distribution hotspots)?
(3) What is the temporal dynamic of biodiversity distribution in the Blue Amazon, considering migratory species?
(4) What natural and anthropogenic pressures can be identified for the Blue Amazon’s biodiversity?
(5) What aspects of the Blue Amazon’s biodiversity have been studied over the years?
(6) What trends and knowledge gaps can be identified in studies on the biodiversity of the Blue Amazon?

Although protocols for systematic reviews exist and are widely recognized as reliable [7,8], the broad scope of the review proposed by INCT-BAA, covering a considerable geographic area and all branches of the tree of life, required adaptations and new implementations. These methodological modifications, which are described in detail in this protocol, were necessary to comprehensively address the research questions outlined above.

## Method details

a) General Method

The review covered the vast majority of studies published on the biodiversity of the Blue Amazon (around 5.7 million km²) until December 2023. The main focus was on data published in peer-reviewed literature using the search platforms Web of Science, ScienceDirect and Google Scholar (Graphical Abstract).

### Eligibility criteria

All peer-reviewed studies that sampled current biological diversity (with taxonomic identification), from microorganisms to megafauna (See on *Search strategies* the full description of studied compartments and working groups (WG)), were eligible, even if the main focus of the study was not to conduct a diversity survey. Studies focusing on past biodiversity (paleontology) were excluded. Only studies published up to December 2023 were included.

Due to the regional importance of the topic, publications were considered in English, Portuguese (to incorporate studies from local Brazilian journals), and Spanish (to include research from Latin American journals).

### Search strategies

The literature search strategies were developed using search strings containing terms related to specific marine biodiversity taxa (a species, class or order, depending on the group) and geographical occurrence in Brazilian marine environments. These terms were searched in the full text (title, abstract, keywords and body of the paper) (Table 1). Only Google Scholar searches were restricted to the titles of the papers due to the generic breadth of Google Scholar's algorithm, which commonly returns many results unrelated to the search string terms. The review was organized into different WGs as follows: WG1: Microorganisms (Virus, Bacteria and Archaea); WG2: Phytoplankton; WG3: Macroalgae and higher plants (Including mangrove); WG4: Zooplankton; WG5: Benthos; WG6: Fish; WG7: Cephalopods; WG8: Sea turtles; WG9: Seabirds; WG10: Pinnipeds-Sirenians-Mustelids and; WG11: Cetaceans. Those responsible for each WG elaborated the search strings (Supplementary File 1), conducted the screening, downloaded the PDFs of the identified literature, and stored them in the *Zotero* reference management software. At the end of this first step, to complement peer-reviewed articles not included in the initial systematic search filters, review studies or studies that used data published by others in addition to their own data were considered during the *backward snowballing process* (see more details below).

b) Step-by-step preparation of Search Terms

**Table 1:** Examples of terms used for the search including a TAXONOMICAL, GEOGRAPHICAL and HABITAT category (following these criteria, new terms were created and applied to each specific Working group (WG).

Taxonomic terms	Geographical terms	Habitat terms
Scientific name of genus or species, in quotation marks: “*Chelonia mydas*”, especially for megafauna. Higher taxonomic level: Copepoda, Teleostei, especially for general WGs like Plankton and Benthos	Brasil*	Oceano, Oceânico, Marinho, Mar
Term that refers to the taxonomic group in different languages: Benthos OR Bentos OR Macroinvertebrados	Brazil*	Ocean* Marine Sea
Popular name of the group in different languages: (“Sea turtle” OR "Tortuga marina)		Marino Oceano Oceánico Mar

Note: The use of * or ? for truncation (e.g. Bra?il or Bra*il, Ocean*) is recommended to expand the scope of searches

The first screening was comprehensive, including the biological group of interest (which can be the scientific name of the taxonomic group [family, genus, species etc.]) and/or the common name of the group in different languages English/Portuguese/Spanish, depending on the case) and place of geographical occurrence (Brazil and/or marine area), using * for truncations. These terms were searched throughout the full article without restricting to title (except for Google Scholar), abstract, or keywords.

Following this initial search, a deeper search was conducted using specific keyword combinations based on the results retrieved in the initial search. This included the identification of possible filter terms (e.g. “coastal”, ‘intertidal’, “wetland” etc.) and exclusion terms (e.g. -reservoir*). Various search strings were applied to retrieve new references and duplicate results (which were quantified) until search saturation was reached, defined as the absence of new results with additional search strings. In the search string, specific words or punctuation marks can be used to narrow or broaden the scope of searches. A glossary of the terms used is available in Box 1.

***First screening:*** ("GROUP" OR "TAXA" OR "Popular Name" in Port/Eng/Esp (if existent)) AND ("Brasil*" OR "Brazil*") AND ("Marinho" OR "Marine")

***Following screenings:*** (“TAXA” OR “Popular Name”) AND (“Brasil*” OR “Brazil*”) AND “VARIABLESTOINCLUDE”) - ‘VARIABLESTOREMOVE’

BOX 1 **Glossary for the developing of research strings**

*AND:* Search for articles with two terms to narrow the search (e.g. Copepoda AND Brazil).

*OR:* Using two different terminologies (e.g. synonyms) broadens the search. It can be useful for searching in multiple languages at the same time, or searching by scientific name and popular name at the same time (e.g.: Phytoplankton OR phytoplankton AND Brazil OR Brazil).

*NOT:* Excludes a term from the search (Science Direct syntax is AND NOT or the addition of the “-” sign followed by the exclusion term in Google Scholar) - e.g.: Phytoplankton AND Brazil NOT (Bacillariophyceae OR diatoms).

*Quotation marks:* (“”) to search for an exact term or phrase (Google Scholar, Web of Science and Science Direct).

*Brackets ():* terms in brackets appear close together to define the priority of the operator or to separate groups without creating ambiguity (Google Scholar, Web of Science and Science Direct).

Asterisk (*): can be used in Web of Science between terms with multiple spellings, for example, with or without a hyphen (meta-analysis, meta analysis; meta*analysis).

Alt-text: Unlabelled box

It should be noted that the search strategies were independent and not general for all the WGs. Thus, each WG used different strings for each search, whether using general or specific terms. The totality of search strings can be found in the Supplementary File 1, and are described in the *Method Validation* section.

c) Snowballing

The snowballing technique refers to the search for relevant works according to previously established eligibility criteria, based on the bibliographic references of other scientific works. These source studies can be literature reviews or studies that have used data already published in third-party studies found in primary searches (e.g. meta-analyses). These papers were then added to Zotero for further data tabulation.

The search for papers using the snowballing technique was limited to one layer. If during the analysis of a paper's reference list another review paper appeared, which could potentially lead to a second snowballing, this second layer of snowballing was not carried out.

d) Storage of the papers selected in the search

Zotero was the platform chosen for storing and organizing the selected papers. This platform was structured with folders organized according to the taxonomic groups: Microorganisms, Phytoplankton, Algae and higher plants, Zooplankton, Benthos, Fish, Cephalopods, Sea turtles, Seabirds, Pinnipeds, Sirenians and Cetaceans.

After searching and screening the results in relation to the eligibility criteria (Graphical Abstract), the studies were added to Zotero. Once each article had been uploaded to the Zotero platform, a second analysis was conducted to verify the eligibility criteria. When a work did not meet these criteria, it was either moved to a subfolder of the group folder or specifically labeled for later quantification of excluded texts.

For each study retrieved, a 'Note' was added to the publication in Zotero. This note included the set of terms used in the search platform (the search string), the total number of articles returned by the search, and the date the search was conducted.

Multigroup folder: Studies that presented data from multiple taxonomic groups (e.g., birds, fish and benthos sampled in a single manuscript) were added to a separate folder (Multigroup) in Zotero. This approach was implemented to avoid possible duplication in the spreadsheet. Articles categorized in this folder commonly involved trophic analyses and broad biodiversity surveys that examined the entire ecosystem. The analysis of these documents was carried out after the completion of work by the other WGs. Studies that had already been entered into spreadsheets within any of the WGs were counted as duplicates.

Note on full text acquisition: In cases where the full text of a publication was unavailable, each WG attempted to contact the corresponding author to obtain the document.

e) Data extraction and tabulation

### Data tabulation

After the search and screening process, all retrieved studies were thoroughly analyzed to extract metadata and biodiversity information — including species, their locations, and times of occurrence. This process involved filling over 70 different data columns from each study. The extracted information and detailed descriptions are provided in Supplementary File 2. This file also contains an extensive glossary of all terms and concepts used not only for the definition of habitats and subhabitats, but also for the identification of study themes and the main drivers influencing biological communities. The definitions were developed by subject-matter experts, and a comprehensive list of references used to support these definitions is provided in the file.

During the data tabulation stage, multiple researchers worked concurrently within each WG. To facilitate coordination and prevent duplication of effort, specific tags were applied to each study in Zotero. Upon being added to Zotero, a study was initially labeled 'Not Added to Table' in red. Once a study was selected for data extraction, its tag was changed to 'Spreadsheeting' in orange, indicating to other team members that its information was being transferred to the state-of-the-art spreadsheets. When data entry was completed, the tag was updated to 'Added to Table' in green.

The data surveyed (arranged in columns) refers to the six guiding questions on the state of the art of Biodiversity in the Blue Amazon (see Introduction). Each WG maintained and completed all the general columns previously defined from the state of the art. However, each WG also added specific columns (Supplementary File 2), taking into account information particularly relevant to their taxonomic group.

### Taxonomic classification

For the extraction of diversity information (i.e., species reported in each study), taxonomic classification followed the criteria below:

• The most recent complete taxonomic classification (from Kingdom to Species) available was used (see below the list of taxonomic platforms used).
• For groups in which the study does not identify at the species level, the lowest taxonomic level identified (Family, Order, Class) was added to the spreadsheet.
• For species whose taxonomic classification has been updated, the current name of the species is inserted and the name used in the article in parentheses “()”. Ex.: *Cyperrus pedunculatus* (*Remirea maritime*).
• For records of hybrid animals, the following nomenclature was used: “Hybrid (Sp1 x Sp2)” (e.g.: Hybrid [*Eretmochelys imbricata* x *Chelonia mydas*]).

## Platforms used for taxonomic classification

• The main source for marine biodiversity in general was WORMS: WoRMS - World Register of Marine Species (https://www.marinespecies.org).

For groups absent from WORMS or that presented some inconsistency (e.g. outdated information), other databases were used:

• General use: GBif (https://www.gbif.org).
• Marine algae and phytoplankton: Algaebase (https://www.algaebase.org) and Flora do Brasil (https://floradobrasil.jbrj.gov.br).
• Microorganisms: National Center for Biotechnology Information (NCBI) (https://www.ncbi.nlm.nih.gov/).
• Marine Mammals: Society for Marine Mammalogy (https://marinemammalscience.org/)
• Seabirds: Birds of the World (https://birdsoftheworld.org/bow/home), Comitê Brasileiro de Registros Ornitológicos (http://www.cbro.org.br/).

*Note on taxonomic classification:* Each WG was composed of specialists in their respective fields, responsible for extracting relevant information and, in particular, for verifying the taxonomy presented in the studies (updating it when species had undergone taxonomic revisions using the platforms described above). However, due to the broad scope of the protocol, species from different taxonomic groups could appear across multiple WGs (e.g., Copepoda species appearing in datasets intended for benthic groups). Therefore, at the end of the data tabulation phase, all occurrences of a group’s focal species found in other WGs datasets were forwarded to the corresponding group leaders for review and correction, if necessary.

## Georeferencing

When georeferencing data for species occurrences and sampling dates were provided by the authors (e.g., presented in a table, supplementary material, or database), they were included as reported. When such data were not directly provided, or when studies presented multiple sampling points, the information was estimated as follows.

General cases

• One average point: When the latitudinal and/or longitudinal scope of the study was < 1 degree (60nm) an average point in the study area (or between the points) were selected.
• Two average points: When the latitudinal and/or longitudinal scope of the study was > 1 degree (60nm) and < 3 degrees (180nm) two average points in the study area (or between the points) were selected.
• Three average points: When the latitudinal and/or longitudinal scope of the study was > 3 degree (180nm) three average points in the study area (or between the points) were selected.
• One average point per Federal state: When studies cover more than one coastal state, at least one point per state was defined.
• An average point of each area, regardless of the longitudinal and latitudinal differences of the study: sample area with habitat change (e.g.: continental shelf, slope and beyond) in which there is no clear distinction in the quantification of diversity between different habitats.

Special cases:

• Stranding and sighting: If the work does not provide the coordinates of the strandings, but only the name of the beaches or the indication of the monitoring section, the coordinate is estimated on the beach; the number of coordinates to be obtained follows the previously mentioned criteria of spatial scope. In the case of strandings, the habitat field receives the value NA;
• Migratory route or displacement: If the work does not provide the dates or coordinates of the displacement of the individuals under study, a starting point of the route and a midpoint are inserted for each Federal state where the animal occurs. It should be noted that if the individual's displacement covers different habitats, the previously mentioned criteria must be followed;
• Acoustics: The geographic coordinates of the position of the acoustic sensor; insert only the coordinates of occurrence, following the criteria previously listed, for global work.

f) Database updating

To ensure the continued relevance of the database generated in this study, regular updates will be conducted every six months following completion of the initial database conclusion. These updates will incorporate newly published literature and any additional species occurrences reported during the intervening period. For each update cycle, a dedicated team will repeat the original search process using all search terms listed in Supplementary File 1, but will limit the search timeframe to publications released since the previous update.

g) Data sharing

All compiled datasets generated during this project will be made publicly available within two years, following the publication of a dedicated data paper and subsequent data papers from each WG. These datasets will be deposited in open-access repositories that support biodiversity data standards and ensure long-term accessibility. Details about data availability, including repository links and access conditions, will be provided on the project’s official website (https://www.bioamazoniaazul.com). To ensure proper credit and traceability, all original data sources will be clearly cited, and metadata will include information on data origin and usage licenses.

## Method validation

**Fig. 2:**
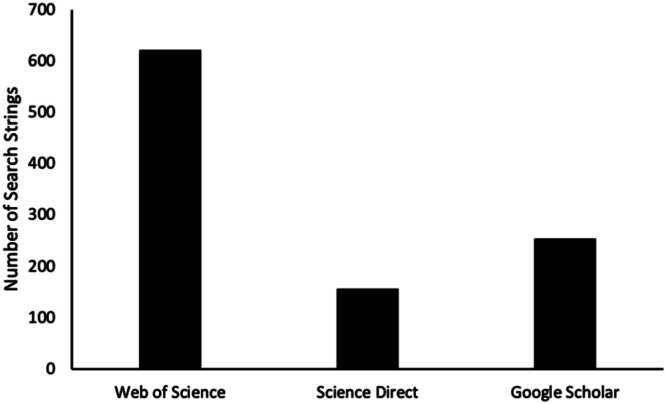
Number of strings for studies acquisition (detailed information in Supplementary file 1).

**Fig. 3:**
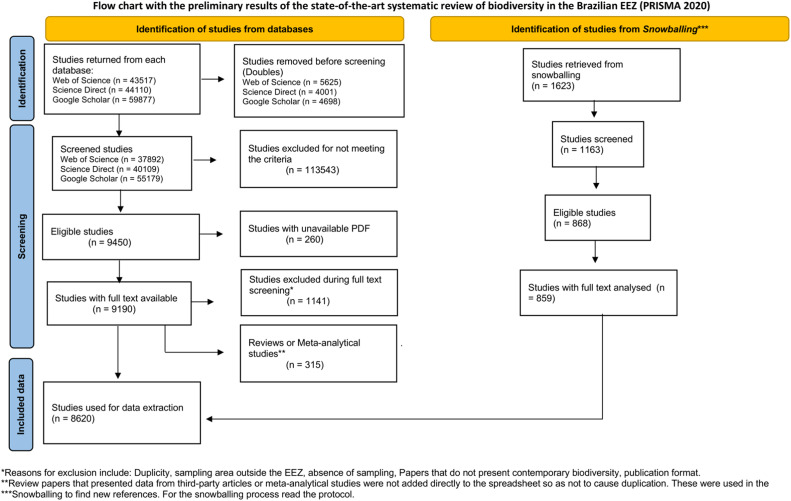
PRISMA 2020 flow diagram. The graphic depicts the method of study selection for this systematic review.

This comprehensive literature review protocol was conducted over the course of one year by a large team of researchers representing all branches of the three of life. During this period, 11 WGs developed and evaluated 1,034 distinct search strings across three platforms: Web of Science, ScienceDirect, and Google Scholar (Fig. 2). Among these, Web of Science accounted for the highest number of search strings, due to its greater volume of results per query and because it did not easily reach saturation (i.e., the point at which no new relevant studies are identified). The search process yielded approximately 147,000 studies (Fig. 3), which were screened according to predefined inclusion criteria (Graphical abstract). Of these, 9,450 met the eligibility criteria for further screening and data extraction.

Only 2.75 % of the eligible studies were excluded due to the unavailability of full-text PDFs—a relatively small proportion that does not compromise the overall robustness of the dataset. Following data tabulation (which remains ongoing for a few WGs), more than 200,000 individual occurrences of over 27,000 different species were identified from 7,687 studies (Table 2). The temporal range of the dataset spans from 1917 to 2023. This protocol therefore provides a robust and representative overview of marine biodiversity research within Brazil's Blue Amazon.

**Table 2:** Overview of the database of each working group, including the number of studies, species identified and the total number of species occurrences extracted.

Working Group	Number of Studies	Number of Species Identified	Total Occurrences
WG1 - Microorganisms	559	5043	15193
WG2 - Phytoplankton	578	2824	15299
WG3 - Macroalgae	1481	3993	18163
WG4 - Zooplankton	1054	3540	23962
WG5 - Benthos	2250	7813	38869
WG6 - Fishes	3066	3265	58747
WG7 - Cephalopods	416	137	2266
WG8 - Sea turtles	501	15 (including hybrids)	9873
WG9 - Seabirds	633	362	20165
WG10 and 10 - Mammals	1276	127	18509

Note: The number of studies does not match the overall total in the state-of-the-art review because studies involving multiple taxonomic groups (e.g., those that investigated fish, plankton, and flora) were utilised by multiple teams, with each team focusing exclusively on their respective groups of interest.

## Limitations

The systematic search was carried out on three different platforms, with variations in the search terms with the aim of incorporating as much published studies as possible. However, old studies are more difficult to detect, as they depend on printed documents having been digitized and made available online. Similarly, many of the paid journals (even with institutional access) make it not possible to access papers, especially the most recent ones. Despite the unavailability of some papers, we believe that this systematic search is very representative of the peer-reviewed literature on the biodiversity of the Blue Amazon. Although a standardized protocol was applied to estimate geographic coordinates for studies lacking precise georeferenced data, some level of spatial uncertainty remains inherent to this approach. Coordinates were often inferred based on the spatial extent described in the study, leading to the use of average points or representative locations. This approximation may obscure fine-scale spatial variation and introduce uncertainty, particularly in cases involving large spatial coverage, habitat transitions, or vague locality descriptions (e.g., general beach names or migratory routes without specific coordinates). Additionally, the use of fixed thresholds (e.g., <1°, 1–3°, >3°) may not fully capture the heterogeneity within study areas. These limitations should be considered when interpreting spatial patterns, especially for analyses sensitive to geographic precision. However, with as large a scope as of this state-of-the-art review covering the extensive Blue Amazon region, we consider that these limitations do not compromise the future findings and interpretations.

## Ethics statements

Not applicable

## Related research article

None

Supplementary material *and/or* additional information

Supplementary File 1 – Search strings

Supplementary file 2 – Readme and Glossary

## CRediT authorship contribution statement

**G.B. Farias:** Conceptualization, Methodology, Writing – original draft. **M. Bassoi:** Conceptualization, Methodology, Resources, Supervision, Writing – review & editing. **J.B. Vasconcelos:** Conceptualization, Methodology, Writing – original draft, Writing – review & editing. **Y. Costa:** Conceptualization, Writing – review & editing. **J.N. Freire:** Conceptualization, Methodology, Writing – review & editing. **J. Lucatelli:** Conceptualization, Writing – original draft, Methodology, Writing – review & editing. **M. Arangüena-Proaño:** Conceptualization, Writing – review & editing. **A.J. Silva:** Conceptualization, Writing – review & editing. **M. Previero:** Conceptualization, Methodology, Writing – review & editing. **L.H. Bordin:** Conceptualization, Methodology, Writing – original draft. **B.P. Ferreira:** Conceptualization, Resources, Project administration. **L.C. Lopes:** Conceptualization, Methodology, Writing – original draft, Writing – review & editing. **E.R. Secchi:** Conceptualization, Resources, Project administration, Writing – review & editing.

## Declaration of competing interest

The authors declare that they have no known competing financial interests or personal relationships that could have appeared to influence the work reported in this paper.

## Data Availability

Data used on the protocol is available as supplementary materials. The Database generated by this protocol will be made available in the next two years

## Appendix Supplementary materials

Image, application 1

Image, application 2
